# Application of "Actor Interface Analysis" to Examine Practices of Power in Health Policy Implementation: An Interpretive Synthesis and Guiding Steps

**DOI:** 10.34172/ijhpm.2020.191

**Published:** 2020-10-13

**Authors:** Rakesh Parashar, Nilesh Gawde, Lucy Gilson

**Affiliations:** ^1^School of Health System Studies, Tata Institute of Social Sciences, Mumbai, India.; ^2^Oxford Policy Management Limited, New Delhi, India.; ^3^School of Public Health and Family Medicine, University of Cape Town, Cape Town, South Africa.; ^4^Department of Global Health and Development, London School of Hygiene and Tropical Medicine, London, UK.

**Keywords:** Actor Interface Analysis, Health Policy Process, Qualitative Synthesis, LMIC Health Policy, Power Systematic Review, Health Systems Research

## Abstract

**Background:** The difference between ‘policy as promised’ and ‘policy as practiced’ can be attributed to implementation gaps. Actor relationships and power struggles are central to these gaps but have been studied using only a handful of theoretical and analytical frameworks. Actor interface analysis provides a methodological entry point to examine policy implementation and practices of power. As this approach has rarely been used in health policy analysis, this article aims, first, to synthesise knowledge about use of actor interface analysis in health policy implementation and, second, to provide guiding steps to conduct actor interface analysis.

**Methods:** We conducted an interpretive synthesis of literature using a set of 6 papers, selected using purposeful searches and focusing on actor dynamics and practices of power in policy experiences. Drawing upon the framework synthesis approach and using a guiding framework, the synthesis focused on 4 questions – the type of actor interfaces formed, the power practices observed, the effect of such power practices on implementation and the underpinning factors for the power practices.

**Results:** Multiple interface encounters and power practices were identified which included domination, control, contestation, collaborations, resistance, and negotiations. The lifeworlds of actors that underpinned the power practices, were rooted in social-organisational power relationships, personal experiences and interests, and social-ideological standpoints like values and beliefs of actors. The power practices influenced implementation both positively and negatively.

**Conclusion:** Based on the learnings from synthesis, this paper provides guiding steps for conducting actor interface analysis. Additionally, it presents 2 useful tools for power analysis: (1) ‘actor lifeworlds,’ to understand underpinning factors for power practices and (2) relationships of lifeworlds, interface encounters and power practices with their effect on policy implementation. We suggest that interface analysis should be applied in more empirical settings and across varied health policy experiences to nuance the method better.

## Background

The implementation gap is often cited to explain the difference between ‘policy as promised’ and ‘policy as practiced’ and remains a key concern in relation to health policy processes in low- and middle-income country (LMIC) settings. Broadly, all policy processes are contingent on wider socio-political contexts and are shaped by the interaction of involved actors, their knowledge and power dynamics, as well as by aspects of decision-making and the policies in question.^1^ A people-oriented approach that considers human agency and attributes at the center of health systems and health policy has, thus, been recognised as important in transforming the practice of health systems and policy.^2^

Power is at the heart of every policy process.^3-5^ Health policy actors use their power and agency to engage in contestation, negotiation and collaboration, giving real-life direction to the policy process. Yet few studies purposefully and fully examine the practices of power applied by policy actors and how they shape implementation. Two recent reviews have highlighted the need for more theoretically diverse studies focused specifically on local practice knowledge around the values and meanings that influence these micro practices of power.^6,7^

One approach to analysis of actor dynamics and practices of power is ‘actor interface analysis.’ Drawn from development studies and rooted in a social constructionist perspective, this approach has been primarily used to investigate social development and practice.^8^ Unlike the frameworks which are often used to define and classify power in health policy literature,^9-12^ actor interface analysis provides a conceptual and methodological entry point to locate the practices of power in actor interfaces and then helps to understand the nature, sources and effect of power practices on policy process. Compared to structural or institutional approaches to analysing and dealing with social problems and development, actor interface analysis additionally seeks to locate actors and their characteristics as central explanations of why the policy process takes a certain shape in each context. It provides an analytic approach to explain how, in similar institutional and structural contexts, the implementation and outcomes of policies can unfold differently,^13^ depending upon actor relations and their power struggles.

Although relevant to the people-centered approaches called for in health systems and policy analysis, actor interface analysis has rarely been explicitly used in understanding health policy implementation, including power practices. Hence, this paper aims first, to synthesise the available knowledge about actor interfaces and related practices of power and their influence over health policy implementation. Second, it seeks to provide guidance for future researchers about how to approach actor interface analysis in investigation of health policy implementation.

In this paper we first outline key concepts underpinning actor interface analysis, before presenting the synthesis method. Our synthesis findings are then presented, followed by discussion of what they indicate about future research needs as well as how interface analysis can be conducted. Our overall intention in this paper is, then, to support the wider application of actor interface analysis in studies of health policy implementation and so better to understand how actors’ practices of power influence implementation. This paper does not seek to synthesise wider evidence on health policy implementation and practices of power, as already presented in some recent reviews.^4,6,14,15^

### Underpinning Concepts of Actor Interface Analysis

Actor interface analysis focuses on examining individual actors and their lived experiences and seeks to explain how the interactions of such experiences with development interventions or policies shape policy practice and outcomes. Norman Long, a developmental sociologist, applied the approach in case studies of agrarian and rural development policies in parts of Africa and Latin America. In a textbook ‘Development Sociology, Actor Perspectives,’^8^ he introduces the actor perspective as* “one that explores how social actors are locked into a series of intertwined battles over resources, meanings and institutional legitimacy and control*.”^8^ Overall, the approach builds on the understanding that policies are not made externally to a local context and then simply implemented as pre-designed blueprints, but instead go through a complex and dynamic process entailing interactions among actors which shape implementation decisions, actions and their outcomes.^1,16,17^

The multiplicity of actors’ lived experiences is called *lifeworlds*, which form the basis for the interaction of actors with each other and with policy interventions within broader implementation contexts. *Lifeworlds* have been described by Long as ‘lived-in’ and largely ‘taken-for-granted’ social worlds. He argues that – *“lifeworlds should be seen as a fluid process in which each individual is constantly self-assembling and re-evaluating his/her relationships and experiences, thereby determining the composition of their lifeworld.”*^8^ The formation of *lifeworlds*is a dynamic process and can be traced back to various contextual aspects of actors’ lives, ranging from knowledge and power relationships in society and organisations, personal characteristics and worldviews influenced by social-cultural-ideological standpoints. We have expanded the elements of these dimensions in Table 1 in presenting the ‘*actor lifeworlds’* framework.

**Table 1:** *“Actor Lifeworlds,”* as Contributors for Socially Constructed Interfaces in a Policy Process^a^

	**Three Broad Dimensions of Actor** ***Lifeworlds***		
	** Relationships of Power**	**Personal Life Concerns or Characteristics**	**Social/ Cultural/Ideological Worldviews**
Characteristic elements for each category	Social positions or status, authority, organisational/ institutional hierarchy, technical/ professional expertise, resourcefulness, gender, caste, class relations	Individual interests, motivation, identity, image, recognition, previous experiences, cognitive and behavioral traits, situations in personal lives, understanding	Values, norms, beliefs, moral standing, religious views, organisational/ institutional norms and culture

^a^ Adapted from Long.^8^

The interaction of actors with each other in relation to a policy alters the course of policy interventions. These points of direct or indirect engagement between actors in relation to a policy intervention can be understood as *actor interfaces*. According to Long, *“interfaces typically occur at points where different, and often conflicting, lifeworlds or social fields intersect, or more concretely, in social situations or arenas in which interactions become oriented around problems of bridging, accommodating, segregating or contesting social, evaluative and cognitive standpoints*.*”*^8^ Actor interfaces are, thus, shaped by the similar, intersecting or differing *lifeworlds* of the actors and are themselves constituted by multiple elements as outlined in Table 1. It is at these sites or interfaces where power struggles among actors are located. Power struggles such as domination, control, collaboration, contestation, resistance or negotiation amongst actors at the interfaces then influence implementation processes and modify the course of action in a way that is distinct from the original implementation blueprint. The categorization of power struggles in this study was based on these practices which is elaborated upon in the results section of this paper.

## Methods

The 4 questions that guided the review presented in this paper were: what type of actor interfaces can develop in health policy implementation? What power practices take place in actor interfaces? What contributes to the formation of interfaces? And what is the effect of these power practices on the implementation process?

**Figure 1:**
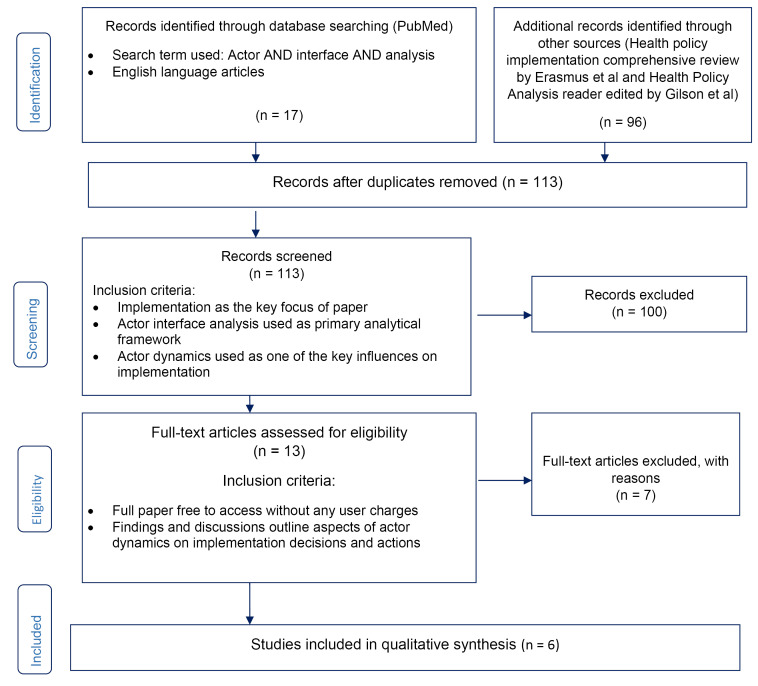
PRISMA Flow Diagram for inclusion of Papers in this Review and Interpretive Synthesis. Abbreviation: PRISMA, Preferred Reporting Items for Systematic Reviews and Meta-Analyses.

The focus of this paper was to understand actor interfaces and *lifeworlds*, and their influence over implementation rather than to synthesise evidence on all facets of implementation. So to answer these questions we conducted a purposive review and interpretive synthesis (which is a type of qualitative synthesis),^18,19^ of relevant studies in health policy implementation from LMICs, published during the period of 1994 to 2017. In order to identify papers to include in this qualitative synthesis, we conducted purposeful searches as illustrated in the Preferred Reporting Items for Systematic Reviews and Meta-Analyses (PRISMA) flow diagram in Figure 1.

The search carried to identify papers for this review was purposeful. We specifically looked for papers reporting LMIC experience only, which offered adequate detail to support interpretive analysis to understand actor relations and power dynamics based on the concepts of actor interfaces. The first author made all the searches, during the period of December 2018 to February 2019. The search included both empirical as well as review papers and duplicates were removed. In the process we used 2 search strategies. First, we searched for published papers related to health policy implementation in LMICs in which the concept of actor interfaces was explicitly applied, without using any time period. A total of 17 results were obtained. Second, we searched for other health policy implementation papers where the focus of study was actor relationships and related power practices, drawing on 2 recently published sources of this body of literature: the implementation section of the 2018 Health Policy Analysis reader^14^ and a previous, comprehensive review paper of LMIC health policy implementation literature published between 1994-2009.^15^ A total of 96 papers were found, including all the health policy implementation papers reviewed within these 2 sources. Grouping the results of 2 searches a total of 113 articles were available. We screened the titles as well as abstracts of these 113 papers, all of which were published between 1994 and 2017. Through the screening process, we selected those papers which had a focus on ‘implementation’ and had examined ‘actor relationships’ or ‘power dynamics’ of actors as key influence on implementation, or which had explicitly applied actor interface analysis (using the terms ‘actor interfaces or actor interface analysis’). A team of 3 persons were involved in discussing the eligibility criteria. However, as the search for papers was focused and purposeful and the eligibility criteria were previously agreed upon, few difficulties were encountered in the screening process. 100 records were excluded in this process which did not fit the eligibility criteria. Out of 13 remaining papers included for full review, only 6 had highlighted actor perspectives and their power relationships as one of the key influences on implementation and they were free to access without any user charges. These 6 papers presented policy experiences from diverse geographies and had different type of policy focus ranging from national to subnational and subdistrict level health policy implementation. These 6 papers were included for this review and synthesis.

We then drew on the framework approach of qualitative synthesis^20-22^ to interpret the information and build knowledge around the 4 key questions of this review. This approach was appropriate as framework synthesis allows the use of a pre identified or predeveloped framework related to theoretical concepts in extracting and coding data deductively.^20-22^ Underpinned by our review of the actor-oriented perspective, we developed a relevant framework that organizes the overarching lifeworld of actors into 3 broad dimensions, each of which is linked to an interrelated set of characteristic elements (Table 1). These actor *lifeworld*dimensions are not, however, mutually exclusive, and may overlap and interact with each other.

The findings and discussion sections from the 6 included papers were analysed using this framework. The extraction template included columns for – short title of the paper and key policy issue discussed, theoretical base used in the study, type of actor interfaces noted, practices of power observed, origin of power practices in 3 broad dimensions of *lifeworlds*and effect of power practices on policy implementation. The data extraction process included coding the extracted data by the 3 lifeworld dimensions and each dimension of the *lifeworlds*were sub-coded for their characteristic elements based on Table 1. This process of synthesis used both the empirical findings presented in the papers as well as authorial judgements about them.^6,15,18^ This approach allowed us to apply the underpinning concepts of actor interfaces to the analysis of the published papers.

## Results

This section presents the findings addressing the 4 synthesis questions identified for this review. The 6 papers reviewed present implementation analyses of a diverse set of health policies in varied LMIC settings. As outlined in Table 2, two papers focused at district level and considered the implementation of a community health worker (CHW) program in South Africa^23^ and a capacity building program for subdistrict level managers and service providers in a southern Indian district.^24^ Other analyses focused on health facility level implementation processes. These included implementation of abortion services in district level health facilities in Ghana,^25^ primary healthcare (PHC) reforms entailing the introduction of a family medicine based model in public health facilities in Bosnia and Herzegovina (BiH),^26^ priority setting and resource allocation in hospitals in Kenya,^27^ and the implementation of a new financial management policy in maternity wards in South African hospitals.^28^

**Table 2:** Observed Actor Interfaces, Practices of Power and Influence on Policy Implementation

**Policy Experience**	**Actor Interfaces Observed**	**Practices of Power Observed and Related Policy Issue**	**Effect of Power Practices**
Implementing abortion policy in health facilities in Ghana^25^	Head of the health facilities (facility managers) and obstetricians	Contestation as facility managers avoided providing abortion services but obstetricians wanted to provide them	Constraining implementation and slow progress on delivery of abortion services
	Service providers (obstetricians and midwives) and service users	Domination of service delivery decisions by providers and negotiation between communities/service users and doctor/nurses about getting abortion services	
	Obstetricians and midwives	Contestation about providing abortion services as midwives avoided providing abortion services and obstetrician wanted to	
	Head of the health facilities and community leaders	Negotiations about the provision of abortion services because health facility managers were to implement the abortion policy, but community leaders did not want them to implement it	
Introduction of family medicine oriented PHC reforms in BiH^26^	Family medicine doctors or GPs and community	Collaboration for the delivery of primary care services	Strengthening implementation and facilitating the policy intent of reforming primary care services
	GPs and specialist doctors	Specialists resisted new model of delivery of primary care services	
	Nurses and hospital managers	Collaboration for delivery of primary care services	
	Nurses and patients	Collaboration for delivery of primary care services	
Implementing new public finance management policy in maternity wards in South African hospitals^28^	Among nurses in maternity wards	Nurses collaborated with each other for reducing consumption of consumable material in wards	Constraining implementation and unintended consequences reflecting in a feeling of frustration, mistrust and disempowerment among nurses, leading to poor quality of maternity services
	Nurses and nursing in charges	Contestation over the use of consumable material in wards	
	Nurses and patients	Contestation over the delivery of pain killers and other medicines	
	Hospital managers and nurses	Domination and control by managers on budgets and consumption of drugs and consumables used for patient care	
Capacity building program for subdistrict level managers and providers, implemented in 2 Talukas, India^24^	Subdistrict administrative area (Taluka) health managers and higher managers	Control of higher managers over decision planning and decision-making processes related to delivery of intended services in Talukas	Better implementation in one Taluka than the other, leading to differential participation in capacity building program and differences in the service delivery performance in the 2 Talukas
	Taluka mangers and service users from community	Taluka managers collaborated with communities for delivery of services in one study area	
	Taluka mangers and service providers	Taluka managers collaborated with service providers for delivery of services in one Talukas	
Implementation of priority setting and resource allocation processes in 2 public hospitals in Kenya^27^	Senior managers and middle level managers	Contestation and negotiations over budget allocations	Constraining implementation in one hospital and facilitating in the other hospital
	Managers and clinicians over participation	Resistance of clinicians to participating in budgetary and planning meetings	
Implementation of a CHW program in a rural South African district^23^	CHWs and clinical managers	Negotiation on the selection of CHWs and CHW payments	Constraining implementation and thinning down of policy intent, except in some cases where CHW recruitment were done and payment were streamlined by some managers
	District managers and provincial managers	Control of provincial managers over the delegation of implementation responsibilities for managing payments of CHWs	
	District managers and clinic managers	Contestation over access to information and budgets	
	Old and new CHWs	Contestation over CHWs recruitment and their roles as well as over receiving stipends	
	Two competing directorates at provincial level	Contestation over resource allocation and payment mechanisms for CHWs	

Abbreviations: GPs, general practitioners; PHC, primary healthcare; CHW, community health worker; BiH, Bosnia and Herzegovina.

The 6 reported policy implementation experiences took place in varied settings and contexts, understanding of which is fundamental to be able to examine the occurrence of actor interfaces and power struggles. As Table 3 summarises, the policies were developed within different national and local level political and health system settings and were implemented in different organisational and interpersonal settings.

**Table 3:** The Contextual Underpinnings of the 6 Reviewed Studies

**Title of the Paper**	**The Underlying Context of the Policy**
Shaping legal abortion provision in Ghana: Using policy theory to understand provider-related obstacles to policy implementation	Ghana had permitted abortion services by law in 1985, but it took a further 20 years for formal abortion policy documents to be publishedby the ministry of health. Even with these documents, the availability of safe abortion services in public hospitals remained limited. In the context of high maternal mortality and with unsafe abortions being one of the leading contributors to maternal deaths in Ghana, this study examined how health system actors interacted to implement the abortion policy in Ghana.
Diffusion of complex health innovations--implementation of primary healthcare reforms in Bosnia and Herzegovina^26^	The study was conducted to understand the implementation of PHC reforms that were based on family medicine practice in the post-civil war period (1992-1995) in BiH. The country’s health system, with a well-developed PHC system with a wide network of publicly financed hospitals and providers, was destroyed by the war. Therefore, a new PHC reform was introduced in 2001, which was based on upgrading the general PHC what? to specialised family medicine care. This reform was implemented alongside other key health system changes, like the introduction of the healthcare and the health insurance law and allowing more decentralised powers for organisation and delivery of health services.
“It makes me want to run away to Saudi Arabia”: Management and implementation challenges for public financing reforms from a maternity ward perspective^28^	This study was nested in the policy arena of reproductive and maternal health programs in the South African context in the post-apartheid era. It sought to understand what affected front line nurses' actions in public hospital maternity wards, in the context of a new PFMA. The PFMA brought in very strict budget and expenditure controls, which were understood by healthcare staff as linked to the possibility of imprisonment or fines for non-adherence. The implementation of this Act was studied in 2 district-level hospitals, which varied by their socio-economic and political contexts. One of the hospitals was in a rich and urban province and the other was in a very poor and rural province of South Africa.
Advancing the application of systems thinking in health: A realist evaluation of a capacity-building programme for district managers in Tumkur, India^24^	A capacity-building program was introduced in one district of a southern state of India in 2009. It aimed at improving the knowledge and skills of the district and subdistrict managers and eventually improving the local health system performance. The training focused on improving planning and supervision capacities of managers, as these processes were centralised and top-down and so, weak at subdistrict levels. The intervention subdistricts (Talukas) varied in terms of the facility environments, subdistrict, and facility-level leadership as well as community demands. The study looked at various factors which affected the implementation of the capacity building program in 2 Talukas.
Actor interfaces and practices of power in a community health worker programme: A South African study of unintended policy outcomes^23^	A new CHW policy, which provided for the training and payment of CHWs was introduced in South Africa around the year 2000. It aimed to upskill already working CHWs and provide them with employment opportunities in the government system. However, in implementation the policy interacted with another scheme, which sought to only train and upskill CHWs (and focusing on their employment), hence focused more on training the younger CHWs. The powers for recruiting CHWs for upskilling and employment were given to the provincial levels, where more than one provincial directorates (directorate of health promotion and HIV/AIDS directorate) competed for program ownership. District level decision-making as primarily in the hands of district and subdistrict managers who formed the interface between the higher-level managers and the CHWs. The study explored the power dynamics of implementing actors and their effect on the course of the policy outcomes in the study area (a subdistrict).
The influence of power and actor relations on priority setting and resource allocation practises at the hospital level in Kenya: a case study^27^	In the devolved system of Kenyan governance post 2013, the central Ministry of Health has policy-making and regulatory roles. At the same time, responsibilities such as allocation of resources and service provision are held by county health systems. The respective counties manage the county-level hospitals. The senior hospital-level committees develop and send the hospital level resource requirements (budgets) to these county offices and allocate the received resources to the hospitals. This study investigated how the power dynamics of actors in the 2 case study hospitals, influenced the resource allocation decisions at these hospitals.

Abbreviations: PHC, primary healthcare; CHW, community health worker; BiH, Bosnia and Herzegovina; PFMA, public financial management act.

### Observed Actor Interfaces and Practices of Power

These papers collectively illuminate the decisions and actions of many types of health policy actors within multiple interfaces formed during implementation of each policy examined (Table 2). The actors considered include the beneficiaries of relevant health services (BiH and Ghana), CHWs (South Africa), midwives (Ghana), hospital nurses (South Africa), district and subdistrict level managers (India and South African CHW program), primary care and specialist physicians (BiH and Ghana) as well as health facility managers and community leaders (Ghana, South Africa, Kenya, BiH and India). Table 2 illustrates that in each policy experience multiple actor interfaces were formed. A variety of practices of power could be observed within these interfaces, which affected policy implementation. We grouped together these practices of power based on the interface characteristics discussed by Long^8^ and founded on the ideas of power practices manifesting in the acts of domination, resistance, and compliance or accommodation drawn from Scott^29^ and Tilly.^30^ Despite the different policy issues examined and the range of actors considered, 3 common practices of power were observed in the interfaces:

1. To control or dominate – observed commonly in relation to some actors holding positional or professional power in the health system eg, hospital managers in South African hospitals, higher level managers in India and service providers in Ghana
2. To contest or resist – seen when some actors objected to or opposed a decision or an action of another actor, as seen in resistance of midwives in Ghana to provide abortion service, or contestation between obstetricians and health facility managers, resistance of specialist doctors to PHC reforms in BiH and resistance of one Taluka manager in India to participate in a capacity building program. While contestation appeared to occur in the form of more opposition between 2 actors engaging in interface encounters, resistance played out more as acts of not subscribing to implementation processes or expectations. For example, in the BiH study, the specialist doctors did not oppose the new family medicine based PHC model directly, but altered their behavior towards the new family medicine specialists (upgraded from general doctors) by not supporting them, which created interpersonal tensions.
3. To negotiate or collaborate with others – negotiations occurred when actors were only partially aligned to an implementation aspect and collaboration entailed supporting an implementation decision or action together. For example, health facility managers in Ghana and influential community leaders negotiated on the provision of abortion services and there were also negotiations across managers on the selection of CHWs and CHW payments in South Africa. In contrast, there were collaboration among general doctors and nurses to implement the new family medicine model in BiH, whilst managers and service providers in one Taluka collaborated to facilitate the implementation of a capacity building program in the Indian experience.

Formation of interfaces was related to specific issues of implementation and often multiple actors had many interface encounters in each experience. For example, in the study from Ghana,^25^ there were interfaces between obstetricians and midwives, and practices of contestation were demonstrated as many midwives avoided providing abortion services while obstetricians were more inclined to deliver abortion services. Similarly, obstetricians and midwives engaged in interface encounters with communities and dominated service delivery decisions when clients sought abortion services. Health facility managers formed interfaces with influential community members as they were under pressure from socially influential people and avoided abortion service provision. In the experience from BiH,^26^ a collaborative exercise of power, which occurred at interfaces between family medicine doctors and nurses, facilitated implementation of new reforms. A family medicine nurse was quoted as mentioning- *‘‘There is a big change in the functioning of the teams; we now work as real teams, all of us: doctors, nurses and patients. There is good co-operation and that helps to deliver better quality services*.*’*’^26^ In contrast, specialist doctors in interfaces with family medicine doctors, resisted the delivery of primary care services through the new arrangement, as they were not happy with general doctors being upgraded to family medicine specialists. In practice this resistance was shown in an altered behavior towards the family medicine doctors. On the other hand, in the South African hospital study, facility managers, maternity ward in-charges and maternity ward nurses engaged in contestations within interfaces formed in relation to the delivery of pain medicines. There were also negotiations between patients and ward nurses over the delivery of these medicines. One nurse mentioned – “*Even when nurses wanted to give pain relief medicines to patients, they felt their hands were tied. They felt they were being not allowed to exercise their moral and professional responsibility*.*”*^28^ Subdistrict managers and district level managers formed interfaces with each other and with facility level service providers over the idea of participating in a capacity building program and the decentralised ownership of programs^24^ in India. Likewise, in the South African CHW program, managers constituted interfaces with CHWs, that were linked to the introduction of new payment mechanisms to CHWs.^23^ There were contestations over resource allocation at the interface encounters of 2 provincial departments as well as of the provincial and district level managers. There were also considerable tensions among 4 subdistrict managers, who undermined each other’s work frequently. Nonetheless, negotiation and collaborations between subdistrict managers and CHWs at times facilitated the program.^23^ Finally, the heads of hospitals, departmental managers and service providers in Kenyan hospitals engaged in contestation over issues of budgetary allocations. On the other hand, the more collaborative interfaces of managers and service providers in one hospital led to a better accepted and comparatively fair distribution of resources.^27^

### How Did Actor Interfaces and Power Practices Affect Implementation of Policies?

Ultimately, the combined effect of the many practices of power exercised in multiple interfaces shaped the implementation course of each policy reviewed (Table 2). Implementation of some of the policies positively benefitted from power exercises such as collaboration and facilitation, while constraining of implementation and a related thinning down of policy intent was seen in other cases related to power struggles of domination, control, contestation, resistance and long-drawn-out negotiations. The impact of power practices varied for each implementation experience. This included unavailability of abortion services due to the resistance shown by midwives and health facility managers in Ghana^25^; the success of family medicine-based primary care reforms in BiH owing to improved motivation and collaboration of family medicine teams^26^; and the varying degree of participation and outcomes of capacity building program in 2 Talukas in India related to feeling of trust, sense of responsibility in one Taluka (better implementation) and a top down control and poor decentralisation of powers in the other Taluka (implementation failure).^24^ Differences in resource allocation and departmental budgets within Kenyan hospitals were linked to sense of mistrust and betrayal among actors in one hospital (unjust resource allocation) and participatory and collaborative work culture in another hospital (a fair distribution of resources).^27^ A better uptake of CHW programs and delivery of payments to CHWs in one South African subdistrict area because of stronger collaboration related to the personal motivation, enthusiasm and sense of responsibility of one subdistrict manager – compared to thinning down of the policy in other areas where tensions of managers and CHWs played out more.^23^ Finally, the unintended effects of a South African financial management policy led to reduced motivation of nurses and poor quality of care in maternity wards which was related to strict top down control and fear of punitive action from higher managers.^28^

### What Underpinned the Practices of Power? Actor Lifeworlds

The *lifeworld*experiences of actors underpin the actor interfaces and the practices of power observed, which are synthesised in Tables 4-6 according to the 3 lifeworld categories identified in the *actor lifeworlds* framework of Table 1.

**Table 4:** Actor *Lifeworlds* Related to Power Relationships

**Policy Experience**	**Observed Power and Knowledge Relationships of Actors Underpinning Practices of Power**
Implementing abortion policy in Ghana	Organisational and social positions of obstetricians as well as the technical expertise of service providers on the clients seeking abortion services
Introduction of family medicine oriented PHC reforms in BiH	Technical expertise on clinical duties of GPs
	Organisational and social positions of specialist doctors, higher technical expertise of specialist doctors on GPs
	Top down control of implementation decisions by managers (hierarchy)
Implementing new public finance management policy in maternity wards in South African hospitals	Strict orders by nursing in-charges for budget control (formal authority and hierarchy)
	Organisational hierarchy, strict budget control, top down orders by health facility managers
Capacity building program for subdistrict level managers, India	Organisational hierarchy, centralised control from district managers on implementation decisions
Implementation of priority setting and resource allocation processes in public Hospitals in Kenya	Organisational hierarchy, decision-making powers, budgetary powers, access to crucial information of managers
	Technical expertise of clinicians
Implementation of a CHW program in a rural South African district	Organisational hierarchy, top- down control over budgets and delegation of duties, access to information
	Organisational hierarchy of provincial managers on district managers and top down command
	Organisational hierarchy

Abbreviations: GPs, general practitioners; PHC, primary healthcare; CHW, community health worker; BiH, Bosnia and Herzegovina.

First, we observed that social and organisational relationships of power and knowledge were a key factor leading to the formation of interfaces, as it is commonly expected. Often top down domination and control was exercised by more socially and organizationally powerful actors, but this was at times resisted by less powerful actors in terms of their hierarchical positions. The reviewed papers offered many examples which could link to this lifeworld category of actors but Table 4 illustrates only some prominently observed examples of this lifeworld category from each policy experience. The most commonly noted power relationships were rooted in organisational hierarchy^24,27^ and the linked chain of management command which was often observed be top down and executed by those policy actors who had higher organizational positions,^24,26,28^ the social positions of influence,^25,26^ control over information or financial resources as well as professional or technical expertise as power to make decisions.^23,26,27^

These relationships of power played out differently across the policy cases. As shown in Table 2, many practices of power across all 6 experiences were rooted in such social-organisational power relationships which are illuminated in Table 4 and discussed in the text below.

For instance, in India, centralised control in the implementation process and resources by the district managers contributed to an interface between the district managers and the service providers in a health facility. The service providers resisted participating in the capacity building program. One PHC service provider noted: “*Nothing much can be done without giving powers at the Taluka level and PHCs. I cannot even appoint a Group D staff. Where is decentralisation in this?*”^24^ In South Africa, maternity ward nurses’ interface with higher managers was characterised by power relations of strict budgetary control and authoritative command of higher managers. Nurses had the feeling of being restricted by poor resource availability and had a fear of punitive action. A nurse mentioned: *“Even when nurses wanted to give pain relief medicines to patients, they felt their hands were tied. They felt they were being not allowed to exercise their moral and professional responsibility*.”^28^ The technical expertise of obstetric doctors put them on interfaces with health facility managers who avoided keeping an explicit provision of abortion services because of another interface of domination from influential persons from community. Here there was a clash of organizational-positional power of health facility managers with technical power of obstetricians. As one obstetrician mentioned: “… *there are too many women dying from unsafe abortion in our hospitals. We have signed on to the MDGs. We must reduce maternal mortality […] It is not as if we don’t know what to do. We know perfectly well what to do! So why are they still dying?”*^25^

Second, a less commonly understood lifeworld underpinning the practices of power was rooted in personal characteristics, concerns or experience of actors individually or collectively, which played a significant role in affecting how actors made their decisions and interacted with others, at various interfaces. Table 5 illustrates some key examples of individual level factors which contributed to formation of interfaces and power practices, which are depicted in Table 2.

**Table 5:** Actor *lifeworlds* Related to Personal Concerns or Characteristics

**Policy Studied**	**Observed Personal Characteristics Contributing to Interfaces and Practices of Power**
Implementing abortion policy in Ghana	Personal image of obstetricians as a clinical decision-maker
	Professional training and identity of obstetricians
	Previous experience of midwives meeting clients who used excuses to get abortion done without actual medical need
	Fear of head of facilities being labelled (image) as abortionists
Introduction of family medicine oriented PHC reforms in BiH	GPs – sense of empowerment and improved confidence to deliver to community expectations
	GPs – expectations of better rewards and salaries, Specialists – sense of insecurity and perceived threat;
	Improved confidence and trust on GPs of nurses in family medicine units
Implementing new public finance management policy in maternity wards in South African hospitals	No incentives for nurses to implement the new finance policy, personal coping mechanisms of nurses
	Patient expectations for pain relief and other medicines
	Fear of punitive action, demotivation and frustration created by punitive action in ward nurses
Capacity building program for subdistrict level managers, India	Previous unpleasant experience of local managers with the higher officers
	Mistrust between higher managers and Taluka managers
	Previous experience of service providers at PHCs about poor financial and human resource management and no actual distribution of powers under decentralised schemes
	Differences of opinions and understanding for the need of services of service providers and managers
Implementation of priority setting and resource allocation processes in public Hospitals in Kenya	Favoring interests of senior managers to some departments; frustration and reduced motivation of middle managers
	Professional identity of clinicians; personal interests of private practice of clinicians
Implementation of a CHW program in a rural South African district	Anger and frustration of CHWs related to previous experiences and new policy
	Difference in energy, enthusiasm and knowledge of new and old managers
	Clinic managers’ understanding of local issues and personal experiences
	Personality traits of some manager – enthusiastic, energetic, experienced in the relevant field

Abbreviations: GPs, general practitioners; PHC, primary healthcare; CHW, community health worker; BiH, Bosnia and Herzegovina.

Some individual factors were noted to be commonly contributing to interface formation across many of the policy cases as shown in Table 5 here and Table 2. For example, the previous experiences of midwives in Ghana, CHWs in South Africa, and managers and providers in India shaped their interactions with other actors and contributed to interface formation. One midwife in the Ghana study had previously experienced that some women wanted an abortion without a medical need, and this influenced her subsequent approach to clients. She mentioned telling one mother:* “I will take your history; examine you, if the pregnancy is truly going to be problematic to you, I’ll refer you. If not, and your reasons are just flimsy, I may counsel you to maintain the pregnancy*.*”*^25^ Similarly in the BiH experience, a sense of personal threat and insecurity among specialists, resulting from empowerment of general practitioners (GPs) as family medicine specialists, led to formation of interfaces between specialists and GPs.^26^ The personal image of obstetricians about their clinical expertise and health facility managers about not getting labelled as abortionists put them in interfaces with others.^25^ The personal characteristics of being an energetic, enthusiastic manager and having relevant experience of her work with a clear vision, meanwhile, allowed a sub district manager in South Africa to provide better training of CHWs, through an interface of collaboration with CHWs.^23^ In contrast, reduced motivation, increased frustration, perception of threat and insecurity among implementing actors led to interfaces with other actors. For example, the reduced motivation and sense of frustration of maternity ward nurses in South African hospitals, that resulted from the fear of punitive provisions under the new act, put them in interfaces with facility managers. Likewise, the specialist doctors in BiH experience, owing to a threat perception and a sense of insecurity, formed interfaces with GPs.^24,26,27^ Personal interest and need for rewards and recognition were noted in BiH experience where family physicians (upgraded GPs) were hopeful for better financial rewards. One GP mentioned: *‘‘We hope that we will have better salaries and that in this way more young doctors will be stimulated to get involved and specialised in family medicine*.*”*^26^ This was also potentially grouped with improved personal image, confidence and professional identity of family medicine teams.

Third, a relatively least explored dimension – the worldviews of actors, influenced by their social-cultural-ideological standpoints, also underpinned the formation of interfaces and power practices, as shown in Table 2. Although, represented less strikingly, compared to the first 2 lifeworld categories above, Table 6 offers some clear examples of this lifeworld category from the policy experiences reviewed. Broadly these were related to – a moral sense of responsibility, religious views, values based in human rights, prevailing cultures and norms of the organisations or health facilities and professional and ethical codes of conduct among others. Most policy experiences included collaborative interfaces with other actors which were related to a sense of responsibility. This was shown by the GP-turned – family medicine specialists in BiH who were more dedicated to deliver services; by service providers in one Taluka from India who were most strongly engaged in the capacity building program, and by some managers in the South African CHW experience who made strong particular efforts to recruitment and upskilling of CHWs.^23,25,26^ On the other hand, in Kenya, prevailing norms within the organisational culture contributed to doctors’ choices and actions in the interfaces with managers, as doctors did not attend resource allocation meetings because it was not a usual practice for doctors. Likewise, the experiences from South African hospitals and Kenya hospitals highlighted that an environment of mistrust and fear contributed to power struggles of actors,^27,28^ as showed in Table 6. Similarly, the resistance of some midwives was also related to their religious views to not conduct abortions and belief in rights of fetus as against the rights of women in Ghana.

**Table 6:** Actor *Lifeworlds* Related to Worldviews Influenced by Social, Cultural, Ideological Standpoints

**Policy Experience**	**Observed Worldviews of Actors Contributing to Interfaces and Practices of Power**
Implementing abortion policy in Ghana	Ethical Code of conduct – Hippocrates oath of obstetricians
	Belief of some midwives in rights of women and of others in rights of fetus
	Personal conflict and dilemma owing to religious and moral views of midwives
	Social stigma and society’s negative outlook towards doctors and facilities which provide abortion services
Introduction of family medicine oriented PHC reforms in BiH	Sense of responsibility, improved self-esteem, morale of GPs
	A general widespread reluctance to change the existing patterns of work of specialist doctors
	Improved sense of responsibility in nurses
Implementing new public finance management policy in maternity wards in South African hospitals	Feeling of compromised professional responsibilities because of not being able to deliver medicines to all patients and sense of frustration among nurses
	Environment of mistrust and fear in health facilities affecting nurses and in charges
Capacity building program for subdistrict level managers, India	Collective sense of responsibility and commitment of service providers in one Taluka because of higher felt need of the local population
Implementation of priority setting and resource allocation processes in public hospitals in Kenya	Atmosphere of suspicion and mistrust in one hospital; value system and belief in decentralised and consultative process of the facility manager in other hospital

Abbreviations: GPs, general practitioners; PHC, primary healthcare; BiH, Bosnia and Herzegovina.

Some examples which are picked from Table 6 and are discussed here, help us in understanding the occurrence of power practices which are discussed in Table 2. In the Kenyan hospital study, a clinician pointed out about a prevailing norm for clinicians to not attend administrative meetings in one hospital: *“To be honest the reason why I have not attended some of these meetings is because I have not seen my fellow colleagues attending the meeting. My senior colleagues…because when you report to a certain institution sometimes you tend to do what people do, you follow the norms*.*”*^27^ Likewise, religious views and moral standing of some service providers against abortion services negatively influenced the abortion policy implementation in Ghana.^25^ For example, one midwife mentioned: “*…I am against abortion because God says keep your bodies as a holy temple for me to come and dwell in you … I wouldn’t want to offend my God. Because he says don’t do it. […] When you do it [abortion], your hands become bloody ….”*In another example from Indian study, a sense of moral responsibility of the service providers and managers, to catering to the pressing needs of the population, in one area of the study contributed to a better commitment and higher service delivery coverage in this area.^24^ A health manager from this Taluka mentioned: *“We felt that we have to do it. So many mothers were just being referred to Tumkur (a higher-level health facility). The delivery load is high and for several months, we had only one obstetrician, but somehow we managed….”* The study from BiH also reports that an overall culture of not being open to take up new challenges or not intending to change the state of affairs, contributed to slow change in PHC reform.^26^ As the authors noted: *“Many informants shared the view that organisations and individuals resisted adoption of the new model and the change in organisational routines it brought, because of ‘reluctance to adopt an innovation,’ ‘inadequate information,’ ‘habit’ or the ‘shock of change.’ Poor communication fed these fears and concerns.”*

## Discussion

This synthesis highlights the nature of power dynamics observed in the interface encounters of actors in the policy experiences reviewed, as well as provides insights about what underpins such practices of power using the construct of actor*lifeworld*s. The findings draw attention to the complex nature of policy implementation, which is heavily influenced by interactions of actors and their power struggles. All 6 policy experiences here demonstrate that interface formation and power dynamics are a result of an interplay of actor *lifeworlds*, which are constituted by interactions of social-organisational and personal contexts of actors. This builds on an actor centric understanding of policy implementation^2,6,15^ and reinforces the notion that actors make meanings of their surrounding realities in relation to policy processes, and illustrates that their day to day decisions and actions are a function of their power struggles.^8^ These struggles can strengthen the implementation and facilitate meeting policy goals as seen in experience from BiH, one Taluka in India and one hospital in Kenya. Power practices may, however, constrain implementation leading to the thinning down of policy intent as seen in experience in the CHW program in South Africa, abortion policy in Ghana and one Taluka in India. Power struggles can also lead to unintended policy consequences as seen in the South African hospital study of financial reforms, where these contributed to worsening environment and poor quality of care in maternity services. Where constraints refer to obstacles that impede implementation, unintended consequences refer to the sorts of impacts that were not envisaged in policy but resulted from how implementation unfolded in practice.

**Figure 2:**
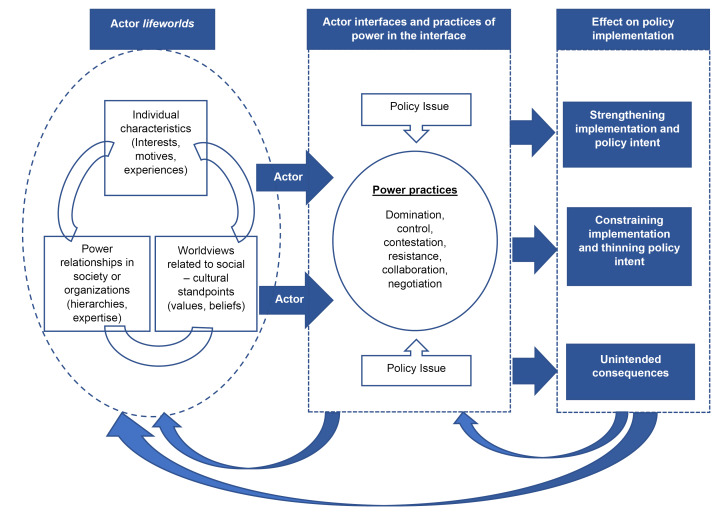
Illustration of Interaction of Actor *Lifeworlds*, Actor Interfaces and Practices of Power and Their Effect on Health Policy Implementation.

These experiences demonstrate the relevance of examining implementation from the actor-oriented perspective and call for more empirical investigations of actor interfaces and power struggles. We have depicted this actor-centric understanding of policy implementation in Figure 2, which shows the relationship among actor *lifewords*, interfaces and power practices, and the consequences for implementation.

This synthesis exercise allowed us to understand the nuances of actor interface analysis as an analytical framework. Our findings suggest that applying an interface lens can be useful in observing actors’ engagement with each other, their routine politics as well as practices of power. This can help policy researchers and practitioners explain the differences between ‘policy as intended’ and ‘policy as practiced,’ as observed in all 6 studies discussed above. However, identification of actor interfaces in health policy processes requires a detailed understanding of the everyday life of actors in their routine contexts as well as of the policy mandate and processes with which actors interact. This is necessary because it is the contextual and lived realities of actors which shape their decision-making in relation to the policy process of focus. The guiding framework used for this analysis, *‘actor lifeworlds’* (Table 1), thus, provides a starting point to examine what underpins the occurrence of interfaces and the related practices of power.

### Summary of Key Steps to Approach Actor Interface Analysis

Guided by our synthesis experience, we have also identified key steps to apply in conducting actor interface analysis, which can help future application of this approach for investigating health policy implementation and power:

- Understanding the relevant policy and its blueprint of implementation: This was a necessary step during our synthesis exercise, as implementation processes as well as interaction of actors were specific to each policy and the implementation setting. This essentially means that researchers should first develop a detailed understanding of what the policy mandate is/was and how it was planned to be implemented in the local context.
- Identification of key actors involved in the policy process: As demonstrated in Table 2, each policy process had many groups of actors who engaged with each other. It is critical to have a comprehensive list of actors involved in implementation and to identify the formal roles in the implementation process of each.
- Identifying actor interfaces and locating practices of power: Actor interfaces can be identified by understanding points of actors’ interactions in relation to a policy component being implemented, as demonstrated in Table 2. At the identified interfaces, practices of power can be considered and can be categorised as, for example, domination, control, contestation, resistance, collaboration and negotiation. While there could be multiple interfaces being formed and re-formed, researchers can focus on those interfaces which have the most significant consequences for the course of policy implementation being studied.
- Analysing effect of power practices: Practices of power at actor interfaces can then be studied to understand their effect on the course of implementation, by analysing how various decisions and actions of actors have affected implementation and policy outcomes.
- Documenting actor *lifeworld*s: This is a critical step to bring meaning and depth to interface analysis. This requires that researchers must seek to understand the lived realities of actors through various qualitative approaches which could include in depth interviews, focus group discussions, observations or ethnographic methods, depending on the purpose, scope and resources available for the investigation. Details of the *lifeworlds* of relevant actors can, then, be documented. The framework provided in Table 1 can be a helpful tool to organise various constituent dimensions (power relationships, personal characteristics and worldviews of actors) and characteristic elements within these dimensions. The examples of actor *lifeworlds* in this synthesis can be seen in Tables 2, 4, and 5. However, these dimensions as well as elements within each dimension are not mutually exclusive and influence each other to affect decisions and action of actors.

As learned during this synthesis process itself, analysts must note that the steps discussed here are not necessarily sequential and analysts may have to use some of these steps in parallel to each other as well as work iteratively between steps in this process.

### Reflections on the Pitfalls and Challenges of Conducting Actor Interface Analysis

Implementing these steps may also face challenges, some of which are specific to conducting an actor interface analysis and some of which are challenges associated with applying a social constructionist or an actor-oriented health policy analysis. In general, researchers must recognize their own positions and biases, as these may affect their interpretations of power dynamics and the underlying lived-in realities of policy actors. It is thus important to draw on relevant theoretical frames in analysing power. Seeing power as a dominant, controlling, or coercive force or ‘power over’ may need to be distinguished carefully from more critically placed or positively construed expressions of power. This includes expressions such as- power to act (power to), power to collaborate (power with) and exercise of inherent individual agency of actors (power within).^12^ Moreover, as Long cautions, in actor interface analysis it is misleading to think of actor interfaces as only interactions of actors or face to face encounters. Rather, it is critical to locate the actor interfaces as socially constructed interfaces that are underpinned by the lived-in social realities of actors, and the policy intervention in question. Without this acknowledgement, actor interactions in the interface encounters may appear as rationally chosen actions (determined by laid down processes), instead of viewed as the result of a continuous assembling and reassembling of social realities by these front-line actors. Finally, in conducting interface analysis one of the biggest challenges is to examine in detail and tease out the lived-in realties or *lifeworlds* of the actors that underpin the power struggles. While an ethnographic approach may help the researchers to see the nuances of the lived realities of actors, other qualitative methods such as non-participant observation and iterative interviewing can help to get a grasp of the predominantly influencing actor *lifeworlds*. Still, the challenge of teasing out lifeworld dimensions, that are clearly demarcated persists and possibly it can be best resolved by recognizing that the various lived realties of actors would overlap and influence each other. Hence, one interface encounter could be underpinned by multiple lifeworld elements.

## Conclusions

In health policy analysis studies, applying actor interface analysis and its concepts can help analysts to study the day to day power struggles of actors and their effect on the policy process more closely. The examination of the lived experiences of actors through the *‘actor lifeworlds’* framework (Table 1) can allow researchers to understand the mechanics behind actors’ decisions and actions, which then can help to inform policy design and implementation keeping actor concerns at the forefront.

The limits of this synthesis also demonstrate the need for further work applying interface analysis in LMIC health policy research. Few papers were identified, despite a purposeful search strategy, that either used interface analysis or provided adequate, relevant detail to be included in the synthesis. It was also not possible in this synthesis to ascertain the degree of impact (or a relative impact) of power struggles on the implementation process. It was also not possible to understand fully the lived realities of actors, given the data available in the papers. Since we used secondary data that was not always based on actor interface analysis, the interfaces were constructed interpretively.

More empirical research is required to understand the ease of use, applicability and benefits of using this approach in relation to other methods that consider an actor oriented focus that is relevant to implementation, such as – stakeholder analysis,^31^ social network analysis,^32,33^ street level bureaucracy^16^ and actor-focused political analyses. We propose that in contrast to stakeholder analysis, for example, interface analysis offers opportunities for a more granular understanding of power practices and their origins, as well as being particularly relevant to understanding implementation experiences. However, given the data available in the papers reviewed, we recognise that more interface analyses are needed to understand what contributes to ‘actor *lifeworlds.’* For example, relatively less studied power relations can also be of high importance to health policy implementation, such as gender, age, duration of working in the health system, access to information etc. Similarly, personal behavioral and cognitive traits of actors and the nuances of actors’ interests and value systems would require a further deep dive into the lifeworld of actors. Such analysis requires an integration of actor-oriented concepts with the realms of psychology, sociology, political sciences, behavioral economics, administration as well as public policy.

Finally, we judge that such analysis can inform policy practice. For example, policy and program strategies could be adapted to harness those *lifeworld*s of actors which appear to influence implementation constructively. Our synthesis suggests that relevant lifeworld aspects include an implementation environment of trust and empowerment, participatory management practices, the moral sense of responsibility, a professional code of ethics, need for recognition and rewards, interest of professional growth and people centric values.

## Acknowledgements

This paper is part of the PhD work of the first author. We acknowledge the mentor team of the Health Policy Analysis fellowship programme that included Dr. Irene Agyepong, Dr. Jeremy Shiffman and Dr. Maylene Shung-king; and all the fellows of the programme for their very useful comments and inputs on the paper during two workshops held in December 2017 and October 2018. We acknowledge guidance from Prof. T. Sundararaman and Prof. Mathew George at Tata Institute of Social Sciences, Mumbai, on various aspects of the research as members of doctoral advisory committee. We also acknowledge inputs of Dr. Zubin Shroff on the final content of the paper. We thank Sharmishtha Nanda for her inputs during the phase of analysis and preparing the paper draft as well as the manuscript.

## Ethical issues

This paper is part of the broader PhD work of the first author, for which the ethical approval was obtained from the Institutional Review Board of the Tata Institute of Social Sciences, Mumbai. As this paper was based on review of the published studies, no separate ethical approval was obtained.

## Competing interests

The authors report support from the Alliance for Health Policy and Systems Research, Geneva, Switzerland, under ‘other’ category, during the conduct of the study.

## Authors’ contributions

RP conducted the literature search, selected papers for review, performed coding and analysis, synthesized the results, and developed the paper draft. NG reviewed paper content and provided inputs. LG mentored the whole process of writing this paper, guided the review and qualitative synthesis as well as provided inputs and reviewed all sections of the paper.

## Authors’ affiliations

^1^School of Health System Studies, Tata Institute of Social Sciences, Mumbai, India. ^2^Oxford Policy Management Limited, New Delhi, India. ^3^School of Public Health and Family Medicine, University of Cape Town, Cape Town, South Africa. ^4^Department of Global Health and Development, London School of Hygiene and Tropical Medicine, London, UK.

## Funding

This paper has been funded through the Health Policy Analysis Fellowship programme, supported by the Alliance for Health Policy and Systems Research, Switzerland.
